# Spinal cord tractography and neuromonitoring-based surgical strategy for intramedullary ependymoma

**DOI:** 10.3171/2023.7.FOCVID2368

**Published:** 2023-10-01

**Authors:** Corentin Dauleac, Sébastien Boulogne, François Cotton, Patrick Mertens

**Affiliations:** 1 Service de Neurochirurgie Fonctionnelle, Hôpital neurologique et neurochirurgical Pierre Wertheimer, Hospices Civils de Lyon;; 2 Université de Lyon, Université Claude Bernard Lyon I, Lyon;; 3 Laboratoire CREATIS, CNRS UMR5220, Inserm U1206, INSA Lyon, Université Claude Bernard Lyon I, Lyon;; 4 Service de Neurologie et électrophysiologie, Hôpital neurologique et neurochirurgical Pierre Wertheimer, Hospices Civils de Lyon; and; 5 Service de Radiologie, Centre Hospitalier Lyon Sud, Hospices Civils de Lyon, France

**Keywords:** spinal cord, intramedullary tumor, tractography, intraoperative neuromonitoring, ependymoma

## Abstract

Because the spinal cord contains a rich concentration of longitudinal and transversal fibers in a very small area, intramedullary surgery could result in a high likelihood of morbidity. In this video, the authors demonstrate the microsurgical technique and surgical skills used to perform excision of an intramedullary ependyma. The authors also present tools (electrophysiology and neuroimaging) that are useful for surgical decision-making and planning, and thus are used intraoperatively, that allow safer and more effective resection of an intramedullary tumor.

## Transcript

This video demonstrates a microsurgical technique for excision of intramedullary tumor at the cervical level (C4–5).

### 0:27 Clinical Presentation.

A 66-year-old woman, without medical history, presented neuropathic pain in the left upper limb for more than 6 months. Neuropathic pain in the left upper limb was felt in the left C5–6 dermatomes. The pain was described as a permanent burning background pain and associated with allodynia (in the same dermatomes). Neurological examination did not find any motor anomaly in the upper and lower limbs. No pyramidal syndrome was observed. However, she had proprioceptive disorders in the left upper and lower limbs, associated with ataxia.

Secondly, symptomatology worsened in a few months: neuropathic pain was no more controlled by pharmacological treatments, and the patient presented sphincter disorders (urinary leakage).

### 1:15 Preoperative Electrophysiological Features.

The patient underwent motor evoked potentials which were normal for the upper and lower limbs. Somatosensory evoked potentials did not find somesthetic segmental response for lower limbs. In fact, no N22 response (spinal cord segmental response) was found, while P39 cortical responses were present. Finally, in order to differentiate peripheric to central alteration, electromyography was performed and showed chronic left C5–6 radicular disorder, without active denervation.

### 1:40 Preoperative Imaging.

Preoperative MRI demonstrated a cystic intramedullary tumor located at the C4–5 levels. Spinal cord edema was observed at the cranial pole of the tumor, and syrinx (from C6 to T2) at the caudal pole of the tumor. Axial images showed that this spinal cord tumor was centromedullary, except at its caudal pole, where it was lateralized to the left. After gadolinium enhancement, the cyst^1^ walls were strongly enhanced.

Finally, diffusion tensor imaging was achieved in order to perform spinal cord tractography.^2–4^ Fiber tracking highlighted complete dilacerations of spinal cord white fibers at the location of the tumor. The standard direction encoded color of tractography was kept to incorporate fiber directionality information: blue color for craniocaudal direction, green color for ventrodorsal direction, and red color for mediolateral direction. At the spinal cord tumor level, as tumor completely dissects spinal cord tracts, the white fibers are displaced, and color-directional encoding accounts for these fibers’ deformations. White fibers were pushed back around the tumor, and no warped fiber was found within the tumor. According to these data from the tractography, intramedullary ependymoma was proposed as diagnosis. So, the scheduled surgical dorsal approach will be confronted with the presence of fibers in the dorsal columns.

According to clinical examination (McCormick scale: II), electrophysiological and MRI features, showing evolutive spinal cord suffering, and before apparition of major gait disorders, microsurgical excision of this intramedullary tumor was proposed and accepted by the patient.

### 3:05 Surgical Approach.

The patient was installed in a prone position with the head fixed with a three-pin head holder. Surgery was performed under general anesthesia without muscle relaxants, to allow the intraoperative neurophysiological monitoring (motor and somatosensory evoked potentials, associated with D-wave recording).^5^

A cervical midline skin incision was performed from C3 to C6 spinous process. A cervical laminotomy was performed from C3 to C5. The superior part of the lamina of C6 was then resected. At this step, an ultrasound machine was used to confirm that this approach was sufficient to control both poles of the tumor.

### 3:45 Dural Opening and Arachnoid Dissection.

The dura mater was opened on the midline. After its opening, it was carefully suspended while being cautious of possible fibrotic adhesion to avoid pulls-up on the spinal cord. Arachnoid mater was opened and then kept intact until the end of the surgery by peripheral suspensions to the dura mater.^6^

### 4:15 Identifying and Opening the Dorsal Median Sulcus.

The posterior side of the spinal cord must be carefully inspected to find the dorsal median sulcus. Because of its tortuosity, the mid-dorsal vein is not always a good anatomical landmark for defining the midline.^6^ Two anatomical elements can facilitate its recognition. Firstly, in case of swollen tumoral spinal cord, the midpoint between left and right dorsal rootlets appears to be a reference for the midline. Secondly, blood vessels running on the spinal cord surface and penetrating into the dorsal median sulcus delineate the sulcus.

Once the dorsal median sulcus was identified, the pia mater was incised using a sharp (ophthalmic) micro knife, and dorsal columns were carefully separated from each other. In order to avoid repeat traumatism on dorsal columns, the pia mater was gently retracted and suspended to the dura, using 8-0 sutures, under somatosensory evoked potentials monitoring.

### 5:14 Microsurgical Resection.

Once the intramedullary tumor was exposed through the opening of the dorsal sulcus, caudal pole of the tumor was highlighted, and cleavage plane must be defined. Once the surgeon had properly analyzed the tumor volume, a microbiopsy was performed for immediate histology analysis of the tumor (because results could influence the surgical strategy), and debulking can begin. It was not necessary to take undue risks for en bloc resection, but it was preferable to achieve a piecemeal resection to avoid repeated trauma on spinal cord white matter.^7^ At this step, ultrasonic aspiration at low intensity could help to reduce the tumor volume. The rule is to avoid any traction or compression, and any kind of mechanical constraint on the normal spinal cord tissue. The tumor was then gently mobilized to highlighted lateral cleavage planes and feeding vascularization was exposed, coagulated (at low intensity), and sectioned. These two steps (debulking and highlighting cleavage plane) were repeated as required, until the cranial pole of the tumor.

The last part of the resection concerned the tumoral ventral pole: it would be performed very carefully, and coagulation of tumoral feeding arteries from the anterior spinal artery should be used with great prudence at this step. With these surgical principles, the goal of the surgery to achieve gross-total resection while maintaining neurological functions can be reached in most of cases.

All the tumoral tissue obtained during surgery was rapidly sent to the laboratory of pathology for tumor cell analysis.

### 6:56 Closure.

Once gross-total resection was achieved and hemostasis controlled inside the cavity (mainly by cotton application and by low-intensity coagulation if necessary), the spinal cord pia mater was closed using 10-0 sutures. Arachnoid leaflets were folded back on the spinal cord in order to avoid local tethered cord syndrome. Then dura mater was closed and reinforced with biological glue.

At the end of the surgery, electrophysiological monitoring demonstrated the persistence of motor and sensory functions: D-wave remained stable during the surgery, and somatosensory evoked potentials were still present at the end of the surgery.

## Disclosures

The authors report no conflict of interest concerning the materials or methods used in this study or the findings specified in this publication.

## Author Contributions

Primary surgeon: Mertens. Assistant surgeon: Dauleac. Editing and drafting the video and abstract: Dauleac, Cotton. Critically revising the work: Dauleac, Boulogne, Mertens. Reviewed submitted version of the work: Dauleac, Cotton, Mertens. Approved the final version of the work on behalf of all authors: Dauleac. Supervision: Mertens. Intraoperative neuromonitoring: Boulogne. Spine tractography: Cotton.

## Supplemental Information

### Patient Informed Consent

The necessary patient informed consent was obtained in this study.
